# Influence of Biofortified Colored Wheats (Purple, Blue, Black) on Physicochemical, Antioxidant and Sensory Characteristics of Chapatti (Indian Flatbread)

**DOI:** 10.3390/molecules25215071

**Published:** 2020-11-01

**Authors:** Anita Kumari, Saloni Sharma, Natasha Sharma, Venkatesh Chunduri, Payal Kapoor, Satveer Kaur, Ajay Goyal, Monika Garg

**Affiliations:** 1 Agri-Biotechnology Division, National Agri-Food Biotechnology Institute (NABI), S.A.S. Nagar (Mohali), Punjab 140306, India; anitaani91@gmail.com (A.K.); sneha.shrma1@gmail.com (S.S.); natasha.micro@gmail.com (N.S.); chundurivenkatesh@gmail.com (V.C.); ssspayalkapur@gmail.com (P.K.); satveerkaur5555@gmail.com (S.K.); 2 Chitkara University School Engineering & Technology, Chitkara University, Himachal Pradesh 174103, India; ajay.goyal@chitkarauniversity.edu.in

**Keywords:** chapatti, black wheat, blue wheat, purple wheat, anthocyanin, antioxidant

## Abstract

Biofortified colored wheat (black, blue, and purple) is rich in anthocyanins and phenolic acid compounds that impart positive physiological effects in humans. A large proportion of wheat is consumed in the form of Chapatti in Asian countries. The effect of chapatti cooking on the proximate composition, bioactive compounds (anthocyanins and phenolics), and antioxidant activities of these wheat varieties were checked in this study. Apart from acceptable sensory parameters, good taste, and soft texture of chapatti, biofortified colored wheat chapatti and flour had higher dietary fibers, protein content, and lower carbohydrate content. Higher soluble and insoluble phenolic compounds, anthocyanin content, and antioxidant activity were in the order of black > blue > purple > white. Chapatti making has reduced their antioxidant activity and anthocyanin content in comparison to flour. Moreover, the reduction in antioxidant activity is less as compared to the decrease in anthocyanin content. Our results suggest that colored wheat can be a better alternative to normal wheat for preparing chapatti as it would have additional health-promoting activities.

## 1. Introduction

Biofortification is the process by which the nutritional quality of food crops is increased in plants during their growth. This can be improved through agronomic practices, conventional plant breeding, or modern biotechnology techniques. Biofortified colored wheat (purple, blue, black) (*Triticum aestivum* L.) is rich in anthocyanins that act as antioxidants [1]. Colored wheat has been reported to have anti-inflammatory activity in a cell line study [1], improve serum antioxidant status and lipid peroxidation in rats [2], improve life span in *Caenorhabditis elegans* [3], control glycemic index in type 2 diabetic human patients [4], reduce total cholesterol, triglyceride, and free fatty acid levels in serum, and improve glucose/insulin resistance in mice [5]. Thus, colored wheat products can be introduced to the daily diet as healthy food.

Bread wheat is an important cereal crop as its flour is processed into several food products such as bread, chapatti, pasta, etc. However, in India, about 75% of the wheat grown is used for the preparation of flat unleavened bread, i.e., chapatti, which is prepared from whole wheat flour, while the remaining is consumed for the preparation of bread, biscuits, cakes, etc. [6]. Chapattis are not only prepared in the Indian subcontinent, but also in the Middle East with a slight variation in the preparation procedure. Chapatti is a diet staple and represents an inexpensive primary source of protein and calories [7]. A chapatti consists mainly of crust and a little crumb. The desired qualities in a chapatti are its higher pliability, puffability, soft texture, light creamish brown color, easy chewiness, and aroma [8]. The quality and nutritional contents of chapatti depend on the variety of wheat used for its preparation. Biofortified colored wheat can serve this purpose to a great extent. Higher antioxidant activity can be an added benefit if it still persists in the final form of chapatti. As chapatti is widely consumed in the Asian region, replacing its starting material from conventional wheat varieties to colored wheat varieties will benefit a huge population in terms of health. So, we aim to assess the antioxidant activities and quality characteristics of colored wheat chapatti in comparison to white wheat chapatti. We provide strong evidence that biofortified colored wheat chapatti contains more health promoting factors, thus making colored wheat an excellent replacement of white wheat for chapatti preparation in order to provide resistance against a wide range of diseases.

## 2. Results and Discussion

**Figure 1:**
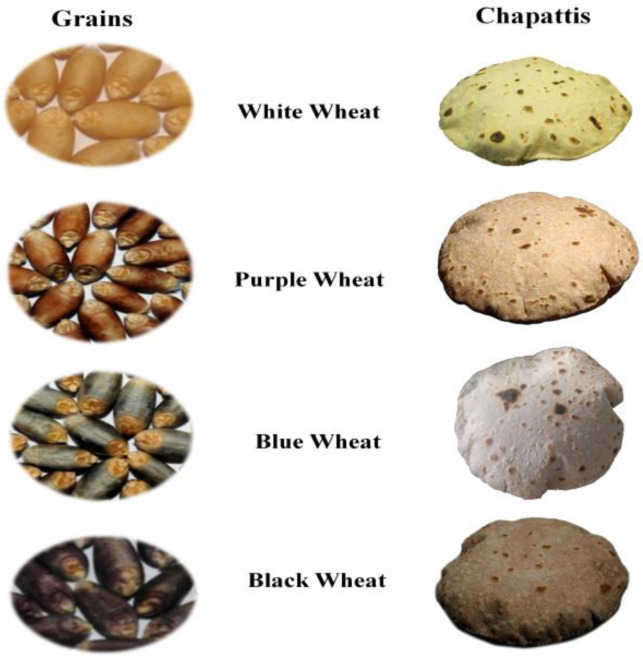
Seeds and chapatti of white and different colored wheat.

Seeds and chapatti of different colored wheat lines are shown in Figure 1. Their detailed physicochemical, antioxidant, and sensory characteristics are as follows.

### 2.1. Grain Characteristics

Wheat kernel weight, size and hardness index are important quality factors for the evaluation of milling and end-use properties of wheat. Among all the colored wheat, thousand kernel weight (TKW) was higher in purple wheat than others indicating bolder seeds (Table 1). The test weight of all the samples remained similar. SKCS grain hardness index varied from 75.07 to 84.91, indicating that all were of the hard type. The highest hardness index was observed in purple wheat (PW) followed by white wheat (WW), black wheat (Bl-W), and blue wheat (BW). Martinek et al. [9] reported higher TKW, lower test weight, and medium hardness for blue wheat. Sodium dodecyl sulphate sedimentation values (SDSS) were higher in white wheat flour (WF) > purple wheat flour (PF) = blue wheat flour (BF) and black wheat flour (Bl-F). Lower SDSS is preferred for good quality of chapatti.

### 2.2. Dough Extensibility and Chapatti Separation Distance

Texture analysis of dough and chapatti plays an important role in determining the quality and acceptability of products by the consumers. Texture analysis of different colored chapattis and dough was determined using a texture analyzer. The present study showed higher dough extensibility in colored wheats (Bl-W and PW) except blue followed by white wheat (Table 1). Dough extensibility is due to the elasticity of the glutenins present in wheat. Previous study has reported higher dough extensibility for blue wheat compared to white wheat [9]. For chapatti making medium strength dough is preferred [8]. Both colored and white wheat doughs had medium extensibility, indicating their suitability for chapatti making. In the case of chapatti, the lower peak load and greater extension distance before rupture is associated with soft and extensible chapatti [9]. The extension distance of black and blue chapattis was higher than the others. It indicated soft and extensible chapatti of Bl-W and BW.

### 2.3. Color Measurement of Flour and Chapattis

WW, PW, BW and Bl-W flours and chapattis were compared in terms of Hunterlab color values (Supplementary Table S1). Higher L * values in WW indicated that WW flour and chapattis were lighter than other wheat flours and chapattis (PW, BW and Bl-W). In case of both flour and chapattis, it indicated a color increase in the order of white < purple < blue < black; same as visually observed. The a * value determines color from green (−) to red (+). The negative a * value in blue flour indicated more green color and a higher positive value indicated higher red color in black wheat flour. The trend of a * value for chapatti was similar to flour, with the lowest value in blue chapatti and highest in black chapatti. The trend observed in flour and chapatti for b * and Chroma value was the same, i.e., white > purple > blue > black, indicating an increasing trend of blue color. Hue value separated purple wheat flour and chapatti from others due to a higher value. A similar trend in flour color value has been reported by Sharma et al. [1].

### 2.4. Sensory Evaluation of Chapattis

The sensory characteristics of chapattis made from different colored whole wheat flours have been shown in Table 2. The overall acceptability scores assigned to chapattis for stickiness, rolling, puffing height, aroma, mouth feel, taste, appearance, and tearing strength were analysed by 10 panelists. Higher water absorption was observed for color wheat chapattis as compared to WW chapatti. Puffing was also higher in colored wheat chapattis. The results indicated that the colored wheat chapattis had similar chapatti scores as observed in WC. This indicates similar acceptance of colored wheat chapatti. The Indian population prefers to consume white wheat chapatti. During the green revolution, high yielding dwarf varieties were introduced in India and Pakistan in 1966. Despite their excellent yield performance, people did not like the chapatti prepared from major cultivated variety Lerma Rojo-64. It had soft red grains that were low in gluten strength [10]. Consumers developed preference for white grain varieties with hard grain and high gluten strength [10]. This led to the selection of new varieties with amber grain color and medium to hard texture. Till now people are associating red color with poor chapatti preference as there is little reference in the literature about soft grain texture of Lerma Rojo-64 except report for CIMMYT [10]. It is observed that the soft grain trait is associated with poor chapatti quality. Therefore, we expect that colored wheat PW, BW, and Bl-W will be liked by consumers as these varieties have a hard grain texture and their sensory evaluation also indicated similar chapatti scores as white wheat chapatti of recipient line PBW621.

### 2.5. Proximate Analysis of Whole Wheat Flours and Chapattis

Proximate composition of whole wheat flour and chapattis has been shown in Table 3. Moisture content of different wheat flours was not significantly different from each other and ranged between 9.96–10.01%. The low moisture content of wheat flour enhances its shelf life by avoiding microbiological growth and other biochemical reaction. Food moisture content is influenced by various parameters like type, varieties, and storage condition [11]. However, among chapattis, a significantly higher moisture content was observed in case Bl-C (39.18%) followed by BC (37.91%) in comparison to PC (34.12%) and WC (33.36%). Moisture content is an important feature of food quality measurement. A higher moisture content will give softness to chapattis.

The protein content of colored wheat flours and chapattis was significantly higher than white wheat flours and chapattis. Protein content in flours followed the order Bl-F (12.14%) > BF (11.13%) = PF (11.01%) > WF (9.93%). The protein contents of chapattis were significantly lower than their respective flours and followed the order Bl-C (10.95%) > BC (9.99%) = PC (9.94%) > WC (8.95%). High protein content in wheat is desirable as it helps in meeting the recommended daily protein requirement and is a key factor in determining the economic value of wheat. Also, the protein content in wheat plays a significant role in determining its end product. The quantity and quality of flour proteins determine the puffiness of chapatti [8]. For chapatti making, a medium protein content is desirable as it provides softness, less tearing resistance, and optimum chewiness to chapattis [7]. Guo et al. [12] found higher protein content (15.17g/100g) in different purple wheat varieties as compared to the white wheat control (13g/100g). Ficco et al. [13] found a higher protein content in yellow durum wheat (14.24%) in comparison to purple durum wheat (13.82%).

The carbohydrate content of WF (67.91%) was significantly higher than that of colored wheat flours (64.43–66.03%). Among chapattis, a significantly higher content was observed in the case of white wheat (47.48%), followed by purple (44.98%), blue (40.93%), and black (38.24%) chapattis. Carbohydrates are rich source of energy and its high content is desirable for meeting daily energy requirements and for making energy-rich breakfast formulations. Our previous study results depicted that the incorporation of colored wheat in the diet lowers body weight gain and improves glucose/insulin resistance in mice [5]. This may be associated with higher anthocyanin and lower carbohydrate contents in these lines.

No significant difference was observed in the fat content of colored and white wheat flours as well as chapattis. The fat content of all flours and chapattis ranged between 1.10% to 1.24% and 0.80–0.85%, respectively. Heating did not cause any significant decline in the fat content of chapattis. Fat content in flours improves the flavor and palatability of the end products. Guo et al. [12] found a higher fat content (1.21%) in purple wheat varieties as compared to the control (0.88%).

Colored wheat flours exhibited higher dietary fiber content than WF. Dietary fiber contents of colored wheat flours (11.85–12.23%) were statistically at par with each other and significantly higher than WF (11.0%). Among chapattis, WC (9.36%) exhibited significantly a lower dietary fiber content than colored wheat flours (10.16–10.78%). Dietary fibers are the carbohydrate polymers that are not hydrolyzed by the endogenous enzymes present in the human body and are considered important as these provide numerous health benefits, such as relieving constipation, maintaining weight, and lowering the risk of diabetes and heart diseases. Guo et al. [12] found no significant difference in insoluble dietary fibers of purple and white wheat varieties. Ficco et al. [13] found a higher total dietary fiber (TDF) content in purple wheat flour (9.8%) in comparison to yellow durum wheat flour (8.4%).

The ash content of flours ranged from 1.67% to 1.88% and within the limit for whole wheat flour (2%). The ash content of chapattis ranged from 1.20% to 1.38%. Colored wheat flours had significantly higher ash content than WF. Among chapattis, Bl-F had significantly higher ash content whereas BF had significantly lower ash content. Kassegn [11] also reported a higher ash content in purple wheat flours compared with refined flours. Generally, ash content is considered as an indicator of mineral content present in food. So, this study suggests that colored wheat could be a better source of minerals than the common white wheat flour. However, a higher ash content negatively affects the texture, taste, pliability, and sheeting properties of chapattis [7].

The proximate composition of colored wheat has been studied by other workers also. Kassegen [11] found that Abyssinian purple wheat flour contained moisture content (10.76%), ash (5.5%), crude protein (8.5%), crude fat (3.03%), crude fiber (7.3%), and carbohydrate (64.85%). Abdel-Aal et al. [14] found moisture content (9.8%), proteins (11.5%), ash (2.1%), and starch (54.4%) in purple pericarp wheat, cv. CDC Prime purple.

### 2.6. Phenolic Compounds and Anthocyanin Content of Flour and Chapatti

**Figure 2:**
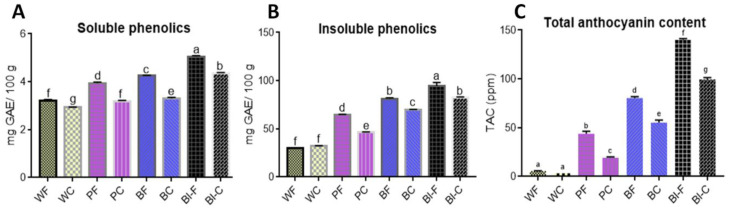
Phytochemical analyses of flour and chapatti samples (**A**) Soluble phenolics content (**B**) Insoluble phenolics content (**C**) Total anthocyanin content of flour and chapatti. All values are expressed as mean ± S.D. Columns labeled with different letters are significantly different (*p* < 0.05). WF-white wheat flour, PF-purple wheat flour, BF- blue wheat flour, Bl-F- black wheat flour, WC-white wheat chapatti, PC-purple wheat chapatti, BC-blue wheat chapatti and Bl-C-black wheat chapatti.

Phenolics are the major secondary metabolites in wheat and play a key role in plant’s pigmentation, growth and reproduction, and imparting antioxidant activity against free radicals [15]. Phenolics have also been reported for anti-allergic, anti-inflammatory, anti-bacterial and anti-cancerous activity [16]. In this study, soluble phenolics content (SPC) of different studied flours samples varied from 3.20 to 5.04 mg GAE/100 g and for chapatti samples 2.93 to 4.36 mg GAE/100 g (Figure 2A). SPC of the anthocyanin-containing flours was significantly higher than white wheat flours (WF). The highest concentration was recorded in black wheat followed by blue and then purple wheat, whereas the lowest was observed for white wheat flour (Figure 2A). Similarly, SPC of colored wheat chapattis was significantly higher than white wheat chapattis (WC). The highest concentration of SPC in case of chapattis was observed in black wheat followed by purple and blue. Lowest SPC was observed in WC. A reduction in the SPC content of chapattis in comparison to flour was 8.4%, 19.1%, 22.3%, and 13.5% for white, purple, blue and black chapattis respectively, with the highest reduction in case of blue wheat. The SPC content decreased in chapattis as compared to the flour with an average of 15.8%.

Insoluble phenolics content (IPC) of flours varied between 29.88 to 94.33 mg GAE/100 g and for chapattis 31.96 to 82.40 mg GAE/100 g (Figure 2B). The order of IPC in flour samples was black > blue > purple > white. Similarly, colored wheat chapattis had higher concentrations than white wheat chapattis. The IPC levels for purple, blue and black chapattis as compared to the flour significantly reduced by 28.42%, 14.12%, and 12.65%, respectively. However, no reduction was observed in case of white chapatti. Sharma et al. [1] reported that colored wheat contains significantly higher SPC compared to white wheat. Meanwhile, Yu and Beta [16] reported an increase in TPC during bread-making in white and purple wheat. This might be due to fermentation in bread making that is not carried out during chapatti making. Li et al. [17] also reported a significant reduction in phenolic content during the processing of purple wheat bran or heat-treated purple wheat bran muffins. Roy et al. [18] have reported a decrease in phenolic content upon cooking in vegetables that may be due to the binding of phenols to proteins, change in the chemical structure, and inactivation of the enzymes by oxidation or inappropriate extraction process. Above all, SPC and IPC of colored wheat chapatti were higher than white wheat chapatti.

Anthocyanins, similar to phenolics, are well-known antioxidants that impart several health benefits [3]. The total anthocyanin concentration in different studied flours varied from 5.42 to 140.08 ppm (Figure 2C). The highest concentration was recorded in black followed by blue and purple wheat, whereas the lowest was observed for white wheat flours. These results were in accordance with the findings of Abdel-Aal et al. [14], Abdel-Aal and Hulc [19], Sharma et al. [1], and Liu et al. [20], who concluded that colored wheat flours had significantly higher anthocyanin contents than white wheat flour. In the case of chapattis, the total anthocyanin content (TAC) varied from 3.31 to 99.44 ppm. The trend followed by chapattis was similar to flour with the order of black > blue > purple > white. A reduction in the TAC content of chapattis was observed as 39%, 56%, 31.3%, and 29% for white, purple, blue, and black chapattis, respectively, with the highest reduction in the case of purple wheat.

The TAC content was decreased in chapattis as compared to the flour with an average decrease of 39%. Other authors have also reported a reduction in anthocyanin content on heating. Abdel-Aal et al. [19] reported the degradation of anthocyanins in blue wheat flour after heating, but degradation was less in comparison to isolated anthocyanins. The protective effect of the food matrix in colored wheat might be saving anthocyanins from degradation during chapatti making. Yu and Beta [15] reported a 55% reduction in anthocyanin content in bread as compared to flour. Pasqualone et al. [21] reported a 57% reduction in anthocyanin content in biscuits made from purple wheat flour. However, it is antioxidant activity that is more important as compared to the phytochemical content and its cooking loss, which is being covered in next section.

### 2.7. Antioxidant Activity of Flour and Chapatti

Free radicals are generated during cellular metabolism or lipid peroxidation and cause the oxidative damage of biological molecules, such as DNA, proteins, carbohydrates, and lipids. These play a significant role in the development of chronic and degenerative ailments, such as coronary heart disease, obesity, aging, autoimmune disorders, and certain cancers [15]. In this study, 2,2′-azino-bis (3-ethylbenzothiazoline-6-sulfonic acid (ABTS), 1, 1-diphenyl-2-picrylhydrazyl (DPPH), and photochemiluminescence (PCL) methods were used for antioxidant activity determination. The antioxidant activities of the soluble, bound phenolic compounds and anthocyanin extracts were analysed in different color wheat flours and chapattis based on their ability to quench radical cation. The extent of discoloration of ABTS and DPPH reagents is used to evaluate the percent inhibition of the ABTS or DPPH radicals by the antioxidants present in a sample.

**Figure 3:**
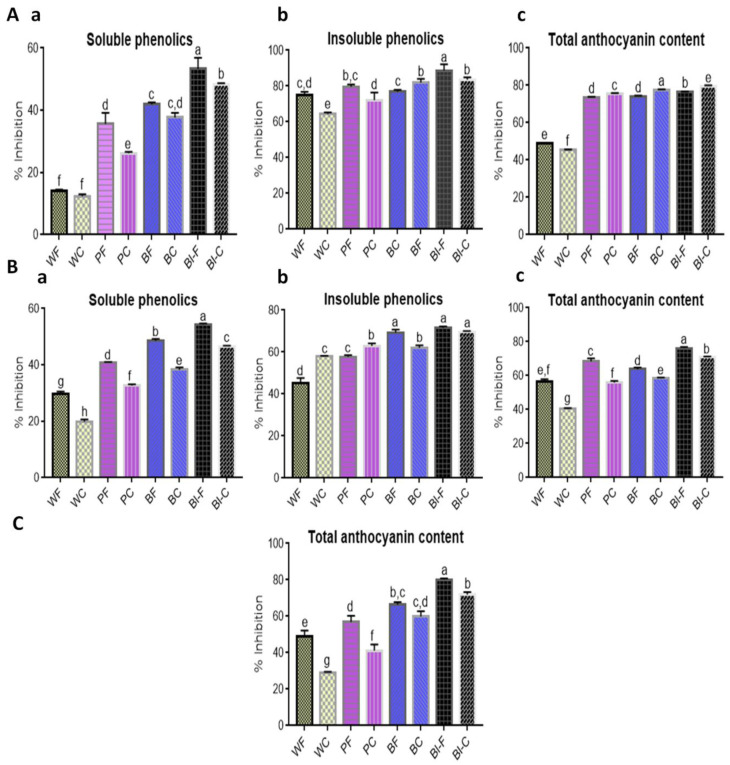
Antioxidant activity of flour and chapatti samples (**A**) 1, 1-diphenyl-2-picrylhydrazyl (DPPH) based antioxidant activity of (a) Soluble phenolics (b) Insoluble phenolics (c) Total anthocyanins of flour and chapatti. (**B**) 2,2′-azino-bis (3-ethylbenzothiazoline-6-sulfonic acid (ABTS) antioxidant activity of (a) Soluble phenolics (b) Insoluble phenolic (c) Total anthocyanins of flour and chapatti. (**C**) Photochemiluminance (PCL) based antioxidant activity of anthocyanin extracts. All values are expressed as mean ± S.D. Columns labeled with different letters are significantly different (*p* < 0.05). WF-white wheat flour, PF-purple wheat flour, BF-blue wheat flour, Bl-F- black wheat flour, WC-white wheat chapatti, PC-purple wheat chapatti, BC-blue wheat chapatti and Bl-C-black wheat chapatti.

In the DPPH assay, antioxidant activities were variable among the colored wheat flours and chapattis. Figure 3A shows the DPPH scavenging activities for soluble and insoluble phenolic compounds. The values varied from 74.98% to 88.64% (flours) and 64.50% to 83.56% (chapattis) for soluble and from 29.56% to 54.31% (flours) and 19.83% to 46.57% (chapattis) for insoluble phenolics. Among the flour samples, the lowest antioxidant activity was observed in white wheat flour. Colored wheat flours exhibited higher antioxidant activity and it was in the same order as TAC and SPC, i.e., black > blue > purple > white. The DPPH levels for white, purple, blue, and black decreased by 12.6% 26.6%, 10%, and 9.2% for soluble phenolics (Figure 3(Aa)) and 14%, 9.3%, 6.1%, and 5.7% for insoluble phenolics (Figure 3(Ab)). Anthocyanin extracts were also analysed for DPPH activity and observed that colored wheat flours and chapattis exhibited higher activity. No decrease in antioxidant activity was observed in anthocyanin extract (Figure 3(Ac)).

Similar trend observed in case of ABTS antioxidant activity, i.e., black > blue > purple > white for flour and chapattis (Figure 3B). The ABTS antioxidant activity levels for white, purple, blue, and black chapattis as compared to flour decreased by 32.93%, 19.81%, 21.14%, and 14.26% for soluble phenolics (Figure 3(Ba)), and 28.7%, 18.5%, 8.3%, and 6.8% for anthocyanin extracts (Figure 3(Bc)). For insoluble phenolics, no reduction was observed in the case of white and purple chapatti, but blue and black decreased by 10.50% and 2.49% (Figure 3(Bb)). Figure 3C shows the PCL based scavenging activities for anthocyanin extracts. The values varied from 48.92% to 80.11% (flours) and 29.21% to 71.87% (chapattis). The antioxidant activity levels were reduced by 27.6%, 9.6%, and 10.3% for purple, blue, and black chapattis, with the highest reduction in purple chapattis.

Many publications have reported higher antioxidant activity of colored wheat compared to white wheat [1,16,21,22,23]. Different authors have reported different trends in the effect of cooking on antioxidant activity. Yu and Beta [16] observed more than 30% higher antioxidant activity of purple wheat bread compared to purple flour. Li and Beta [23] observed a higher antioxidant activity of purple wheat bread. Pasqualone et al. [21] observed more than 15% increases in the antioxidant activity of biscuits prepared from purple wheat. Li et al. [22] reported a decrease in the antioxidant activity of purple and black wheat noodles and steamed bread as compared to flour. Therefore, it depends on the method used for product making and extraction of anthocyanins from the flour and finished products. Alavi et al. [24] reported an increase in the antioxidant activity of extruded products with apple and tomato pomace, despite a decrease in antioxidants (phytochemicals). Thus, most of these studies have indicated that, although there is a decrease in anthocyanin content during heating and product making, there is still quite less of an increase or reduction in antioxidant activity compared to the reduction in anthocyanin content. The reason for this might be an increase in total phenolic content, and another hypothesis may be that the breakdown product of anthocyanins after heating might be causing higher antioxidant activity than their colored and glycocylated forms.

## 3. Materials and Methods

### 3.1. Plant Material

Plant material included one white wheat line (PBW621) and three advanced colored wheat lines (purple: NABIMG-10, blue: NABIMG-9, black: NABIMG-11) selected from backcrossed filial generations (BC1F10) of a cross between recipient white and donor black wheat lines. The donor black wheat developed by crossing blue colored, 4E Ag. elongatum chromosome substitution line Shou Ien 4E(4D) with purple-colored mutant line, was procured from Dr. Hisashi Tsujimoto, Arid Land Research Center, Tottori University, Tottori, Japan. Recipient line, PBW621, was high yielding, disease-resistant and locally adapted cultivar. They were grown in the farms of National Agri-Food Biotechnology Institute, Mohali, Punjab in the main season and in the farms of off-season nursery facility provided by the Indian Institute of Wheat and Barley Research at Keylong, Himachal Pradesh, India, in the offseason.

### 3.2. Preparation of Wheat Flour

Wheat grains were thoroughly cleaned to remove any dirt, dust, insect excreta/feathers or admixture of other food grains. The clean graded materials were grounded in the electric grinder to make whole wheat flour and sieved by 0.5 mm mesh sieves. The flour samples obtained were kept in an air-tight container before use.

### 3.3. Physico-Chemical Properties of Wheat

Grain hardness index (GHI), diameter and thousand kernel weight (TKW) were analysed with a single kernel characterization system (SKCS) using the American Association of Cereal Chemists (AACC) method 55-31 (2000). The weight, diameter and hardness were recorded for 300 seeds of each sample and TKW was calculated. Test weight or hectoliter weights of all the samples were determined using test weight module incorporated in near-infrared spectrophotometer (NIR- spectrophotometer) (Foss, Hilleroed, Demnark) [25]. Sodium dodecyl sulphate sedimentation (SDSS) was determined by Garg et al. [26].

### 3.4. Chapatti Preparation

Chapattis were prepared according to Kadam et al. [27] with slight modifications. Briefly, different colored wheat flours were used to make the chapattis after mixing with suitable water as mentioned in Table 2. All the ingredients were hand-kneaded to make the dough and left for 30 min at room temperature (25 ± 1 °C). Then, 40 g dough was rolled up manually in round sheets. Chapattis were baked at 210 °C, allowed to cool down, and stored in sealed pouches. For each replicate, chapattis were freshly prepared and separately stored. Subsequently, 3 chapatti/replicate of each variety were used in each experimental analysis.

### 3.5. Proximate Analysis of Flours and Chapatti

Proximate analysis was conducted on the whole wheat flours and different chapattis according to Association of Official Analytical Chemists [28] standard methods. Samples were analysed for moisture using MA35 Sartorius Moisture Analyzer (Sartorius Weighing Technology GmbH, Goettingen, Germany), which was set at a temperature of 150 °C and heated for 30 min. Crude fat analysis was conducted based on Soxhlet Extraction Method [28]. Ash content analysis was carried out based on dry ashing method by incinerating samples in a muffle furnace at 550 °C to white ash [28]. Crude protein analysis was determined based on Micro-Kjeldahl Method [28]. The dietary fiber content was determined using the protocol described by Lee et al. [29] with slight modifications. Briefly, defatted 1 g flour and chapatti samples were suspended in 40 mL of MES-Tris buffer and treated with 150 µL of heat-stable α-amylase at 95 °C for 30 min and then digested with 150 µL of a 50 mg/mL protease solution at 60 °C for 30 min, followed by incubation with 200 µL of amyloglucosidase (60 °C, 30 min) to remove protein and starch. After that, 95% of preheated ethanol was added to the hydrolysate and allowed to precipitate. Total volume was passed through the sintered glass crucibles using the Fibertec E system. The retained fiber was oven-dried at 105 °C, and weighed. Protein and ash were determined and deducted from fiber residue for weight correction. Carbohydrate content was calculated from moisture, crude fat, ash, and crude protein contents as follows:

% Carbohydrate = 100% − (Moisture + Crude fat + Ash + Crude protein) %

### 3.6. Physical Characteristics of Chapattis

#### 3.6.1. Texture and Dough Extensibility

Dough extensibility and the textural property of chapatti were measured by using a texture analyzer TATX2 Texture Analyser (Stable Microsystems, Godalming, UK) [30].

#### 3.6.2. Color Analysis

The color of the whole wheat flour chapattis was evaluated by using a ColorFlex EZ Spectrophotometer 171 (Hunter Lab Inc., Virginia, WV, USA). The colorimeter was calibrated with a standard white and black plate. The color of samples was recorded in terms of L *, a * and b *. The L * values determine lightness from black (0) to white (100), a * value determines color from green (−) to red (+) and b * value determines color from blue (−) to yellow (+). Chroma, an expression of the purity or saturation of a single color, was calculated by using (a *^2^ + b *^2^)^0.5^. Hue value measures the most obvious value of a color, and was calculated by using tan^−1^(b */a *)^2^ [26].

#### 3.6.3. Sensory Evaluation of Chapatti

Sensory evaluation of chapattis was carried out by 10 trained panelists by assigning score for dough stickiness (10), chapatti rolling (10), puffing height (10), aroma (10), mouth feel (10), taste (10), appearance (10), tearing strength (10) and after one day of storage at 25 °C for chapatti rolling (10), puffing height (10), taste (10) and tearing strength (10). The overall quality (120) was taken as the combined score of all twelve previous attributes and averaged to get a 10-point score according to Gurushree et al. [31] with modifications as mentioned above.

### 3.7. Determination of Phytochemical and Antioxidant Activity

#### 3.7.1. Soluble Phenolic Content

Total phenolics were determined using the Folin–Ciocalteau reagent [32]. Samples (2 g) were homogenized overnight in 80% aqueous methanol at 125 rpm at room temperature and centrifuged 10,000× *g* for 15 min and the supernatant was saved. The residue was re-extracted twice with 80% methanol and supernatants were pooled and evaporated in a rotatory evaporator. The residue was dissolved in 5 mL of methanol. 100 µL of this extract was diluted to 1 mL with water and 1.5 mL of Folin–Ciocalteau reagent was added. After 3 min, 1.5 mL of 7.5% of sodium carbonate was added and the contents were mixed thoroughly. Absorbance was measured at 650 nm in a UV spectrometer after 60 min. The results were expressed as mg gallic acid/100 g of fresh weight material.

#### 3.7.2. Insoluble Phenolic Content

The residue collected after 80% methanol extraction was subjected to alkaline hydrolysis using the method described by Yu and Beta [16] with some modifications. First, 15 mL of 10M NaOH was added to the dried residue and hydrolyzed on a shaking water bath (for 4 h at 25 °C). The hydrolyzed mixture was adjusted to a pH between 1.5 and 2.0 with 6N HCl and then extracted three times with ethyl acetate. Ethyl acetate was separated from the aqueous layer by centrifuging at 2500× *g* and 4 °C for 5 min. The supernatant was collected and evaporated to dryness at 35 °C in a rotatory evaporator, reconstituted in 5 mL 50% methanol (*v/v*), filtered through a 0.22-µm nylon syringe filter, and stored at −20 °C. The extract was used for the determination of bound phenolic content, DPPH scavenging activity, and ABTS+ decolorization capacity.

#### 3.7.3. Total Anthocyanin Content

Anthocyanins were extracted according to the method described by Young and Abdel-Aal [33]. Acidified methanol (85 mL Methanol: 15 mL, 1N HCl, *v/v*) was used for the extraction of anthocyanins. Extracts were stored at −20 ˚C overnight for precipitation of large molecules and filtered through a syringe filter of 0.45 µm pore size. Absorbance was measured at 535 nm against acidified methanol as blank. TAC was calculated as cyanidin 3-glucoside and expressed as mg/Kg:

C = (A/e) × (V/1000) × MW × (1/sample wt) × 106

where C is the concentration of total anthocyanin (mg/kg), A is the absorbance of sample, e is molar absorptivity of cyanidin 3-glucoside (25,965 cm^−1^M^−1^), V is the total volume of anthocyanin extract used, and MW is the molecular weight of cyanidin 3-glucoside (449).

#### 3.7.4. DPPH Radical Scavenging Activity

The antioxidant activity of the methanol extract was measured based on the scavenging activities of the DPPH radical. 100 µL of wheat extract (80% methanol) was added to 3.9 mL freshly prepared methanolic DPPH (60 µmol/L) solution. After 30 min of incubation period in the dark, at room temperature, the absorbance was measured against a blank (methanol) at 517 nm [16]. The inhibition of free radical DPPH in percent (%) was calculated using the formula:

Percentage inhibition (%) = [(A_control_ − A_sample_)/A_control_] × 100

where A_control_ is the absorbance of the control reaction (containing all reagents except test compound) and A_sample_ is the absorbance of the test compound. All tests were carried out in triplicate.

#### 3.7.5. ABTS Radical Cation Decolorization Assay

The ABTS+ reagent was made by mixing ABTS stock solution (7 mM) (38.4 mg ABTS in 10 mL distilled water) and potassium persulfate stock solution (2.6 mM) (7 mg potassium persulfate in 10 mL distilled water) at a ratio of 1:1 for 2 min vortex. The mixture was stored for 12 to 16 hrs in the dark at room temperature before use. Then, 1 mL of ABTS+ reagent was diluted with distilled water and absorbance at a wavelength of 734 nm was adjusted to 0.9 ± 0.02 by diluting the solution further with methanol. The antioxidant activity of soluble phenolic extracts and anthocyanin extracts (100 μL) was estimated by adding 3.9 mL of freshly made ABTS+ reagent [16]. The absorbance was determined at t = 30 min.

Percentage inhibition (%) = [(A_control_ − A_sample_)/A_control_] × 100

where A_control_ is the absorbance of the control reaction (containing all reagents except test compound) and A_sample_ is the absorbance of the test compound. All the tests were carried out in triplicate.

#### 3.7.6. PCL Assay

The antioxidant capacity of extracts was determined using a Photochemiluminance instrument (Analytik Jena, Leipzig, Germany), by using a water-soluble substances kit (ACW) as per the manufacturers protocol. All tests were expressed as % inhibition activity according to Sharma et al. [1].

### 3.8. Statistical Analysis

Results were expressed as means ± standard deviation (SD) and based on dry weight (DW). The results were submitted to variance analysis (ANOVA) and Tukey test (5% probability) using the software IBM SPSS 21.0 (IBM, Armonk, New York, NY, USA) and Graph Pad Prism 7 (GraphPad Software, San Diego, CA, USA).

## 4. Conclusions

Wheat and wheat products are important staple foods that are consumed worldwide. This study highlights that colored wheat varieties constitute an excellent source of fibers, proteins, phytochemicals, and impart a higher antioxidant potential. So, the consumption of colored whole grains as part of the diet is recommended for health reasons. Notwithstanding, colored wheat varieties contained all the features required for commercial product development, paving the way for their industrial utilization.

## Figures and Tables

**Table 1:** Quality parameters of colored wheat.

Sample	Grain Hardness Index	Kernel Diameter (mm)	TKW (g)	Test Weight (Kg/hl)	SDSS (cm)	Chapatti Separation Distance (mm)	Dough Extensibility	
							Force (gm)	Distance (cm)
WW	76.22 ± 2.15 ^a^	3.04 ±0.06 ^b^	45.18 ± 2.00 ^a^	1191.5 ± 16.71 ^a^	8.96 ± 0.15 ^a^	7.81 ± 1.59 ^a^	27.11 ± 4.27 ^a^	1.84 ± 0.23 ^a^
PW	84.91 ± 0.41 ^b^	3.18 ± 0.02 ^c^	49.29 ± 0.86 ^b^	1192.39 ± 0.68 ^a^	7.73 ± 0.21 ^b^	8.72 ± 1.87 ^a^	36.67 ± 5.65 ^b^	1.91 ± 0.3 ^a^
BW	75.07 ± 0.48 ^a^	2.77 ± 0.01 ^a^	43.55 ± 0.60 ^a^	1174.13 ± 1.10 ^a^	7.43 ± 0.12 ^b^	11.36 ± 1.53 ^b^	20.61 ± 4.51 ^c^	1.18 ± 0.08 ^b^
Bl-W	76.12 ± 0.71 ^a^	2.74 ± 0.01 ^a^	42.7 ± 0.50 ^a^	1171.76 ± 0.77 ^a^	6.63 ± 0.32 ^c^	11.21 ± 1.73 ^b^	31.33 ± 2.5 ^a^	1.49 ± 0.14 ^c^

WW-White wheat, PW-Purple wheat, BW-Blue wheat, Bl-W-Black wheat. All values are expressed as mean ± S.D (n = 6). Different letters in the superscripts in each column are significantly different (*p* < 0.05) with values designated with a < b < c.

**Table 2:** Sensory evaluation of colored wheat chapattis.

Parameters	White Flour	Purple Flour	Blue Flour	Black Flour
**After Cooking**				
Stickiness	8.70 ± 0.30 ^a^	8.20 ± 0.30 ^a^	9.50 ± 0.50 ^b^	8.17 ± 0.29 ^a^
Water (mL)	165.0	180.0	185.0	185.0
Roll out Easy (1–10)	10.0 ± 00 ^c^	7.30 ± 0.60 ^a^	9.00 ± 0.00 ^b^	9.50 ± 0.50 ^b,c^
Puffing Height (10)	6.80 ± 0.70 ^a^	8.30 ± 0.40 ^b^	8.67 ± 0.41 ^b^	8.92 ± 0.38 ^b^
Aroma (10)	8.40 ± 0.50 ^b^	8.30 ± 0.50 ^a,b^	8.11 ± 0.55 ^a,b^	7.61 ± 0.86 ^a^
Mouth feel (10)	8.80 ± 1.10 ^a^	8.50 ± 1.10 ^a^	8.83 ± 0.61 ^a^	8.06 ± 1.24 ^a^
Taste (10)	8.60 ± 1.50 ^a^	8.50 ± 0.90 ^a^	8.56 ± 1.33 ^a^	7.83 ± 1.54 ^a^
Appearance (10)	8.00 ± 1.20 ^a^	8.10 ± 1.00 ^a^	8.11 ± 0.74 ^a^	7.94 ± 0.63 ^a^
Tearing strength (10)	8.00 ± 1.60 ^a^	8.10 ± 0.70 ^a^	8.33 ± 0.66 ^a^	7.78 ± 0.71 ^a^
**After 1 day**				
Roll out (10)	7.90 ± 0.30 ^b^	7.10 ± 0.30 ^a^	8.25 ± 0.29 ^b^	8.13 ± 0.25 ^b^
Puffing Height (10)	5.30 ± 0.50 ^a^	8.10± 0.30 ^c^	8.13 ± 0.25 ^c^	6.88 ± 0.63 ^b^
Taste (10)	7.80 ± 0.30 ^a^	7.80 ± 0.30 ^a^	7.75 ± 0.29 ^a^	7.75 ± 0.50 ^a^
Tearing strength (10)	7.10 ± 0.30 ^a,b^	7.60 ± 0.50 ^b,c^	7.88 ± 0.25 ^c^	6.50 ± 0.41 ^a^
Chapatti Score	7.90 ± 1.20	8.00 ± 0.40	8.40 ± 0.50	7.90 ± 0.80

All values are expressed as mean ± S.D (*n* = 10), where n stands for no of panelists. Different letters in the superscripts in the same row are significantly different (*p* < 0.05) with values designated with a < b < c.

**Table 3:** Proximate analysis of colored wheat and chapattis.

S. No	Carbohydrate	Moisture	Fat	Protein	TDF	Ash
WF	67.91 ± 0.39 ^a^	10.07 ± 0.31 ^d^	1.10 ± 0.21 ^a,b^	9.93 ± 0.11 ^c^	11.02 ± 0.45 ^b^	1.67 ± 0.29 ^a,b^
PF	66.03 ± 0.64 ^b^	9.96 ± 0.22 ^d^	1.15 ± 0.14 ^a,b^	11.01 ± 0.02 ^b^	11.99 ± 0.37 ^a^	1.88 ± 0.2 ^a^
BF	65.80 ± 0.29 ^c^	10.01 ± 0.19 ^d^	1.21 ± 0.19 ^a^	11.13 ± 0.1 ^b^	11.85 ± 0.35 ^a^	1.83 ± 0.29 ^a^
Bl-F	64.43 ± 0.61 ^c^	9.96 ± 0.24 ^d^	1.24 ± 0.26 ^a^	12.14 ± 0.27 ^a^	12.18 ± 0.4 ^a^	1.83 ± 0.29 ^a^
WC	47.48 ± 1.04 ^d^	33.36 ± 1.05 ^c^	0.85 ± 0.03 ^a,b^	8.95 ± 0.07 ^d^	9.36 ± 0.13 ^d^	1.25 ± 0.02 ^b^
PC	44.98 ± 0.34 ^e^	34.12 ± 0.31 ^c^	0.80 ± 0.02 ^b^	9.94 ± 0.07 ^c^	10.16 ± 0.11 ^c^	1.25 ± 0.06 ^b^
BC	40.93 ± 0.52 ^f^	37.91 ± 0.28 ^b^	0.84 ± 0.02 ^a,b^	9.99 ± 0.31 ^c^	10.33 ± 0.07 ^c^	1.2 ± 0.00 ^b^
Bl-C	38.24 ± 0.64 ^g^	39.18 ± 0.6 ^a^	0.85 ± 0.01 ^a,b^	10.95 ± 0.39 ^b^	10.78 ± 0.08 ^c^	1.38 ± 0.14 ^a,b^

WF-white wheat flour, PF-purple wheat flour, BF-blue wheat flour, Bl-F- black wheat flour, WC-white wheat chapatti, PC-purple wheat chapatti, BC-blue wheat chapatti and Bl-C-black wheat chapatti and TDF-total dietary fiber. All values are expressed as mean ± S.D (*n* = 3). Different letters in the superscripts in each column are significantly different (*p* < 0.05) with values designated with a < b < c. All values were given in percentage (%).

## Supplementary Materials

The following are available online.
