# Supplementary material for: Influence of Biofortified Colored Wheats (Purple, Blue, Black) on Physicochemical, Antioxidant and Sensory Characteristics of Chapatti (Indian Flatbread)
Source: Molecules. 2020 Nov 1;25(21):5071. doi: 10.3390/molecules25215071 (PMC7663450; doi:10.3390/molecules25215071)
Supplement: Supplementary file 1 [file molecules-25-05071-s001.pdf]

Supplementary table 1: Hunter lab color (L*, a* and b*) values of different color flours and chapattis.

Sample	L*	a*	b*	Chroma	Hue
			**Flour**		
**WF**	84.57±0.28^{a}	1.57±0.05^{a}	11.93 ± 0.17^{a}	12.03 ±0.18^{a}	0.11±0.15^{a}
**PF**	78.43 ± 0.3^{b}	2.73±0.07^{b}	9.65 ±0.17^{b}	10.03±0.19^{b}	5.71 ± 0.8^{b}
**BF**	79.7 ± 0.27^{c}	-0.05±0.03^{c}	7.22 ± 0.13^{c}	7.22 ± 0.13^{c}	1.52 ± 1.1^{c}
**Bl-F**	74.66±0.12^{d}	7.22±0.13^{d}	5.98 ± 0.06^{d}	6.2 ± 0.06^{d}	2.84±0.64^{d}
			**Chapatti**		
**WC**	63.44 ± 1.4^{e}	4.36 ±0.24^{d}	21.82 ±0.75^{e}	22.26 ± 0.78^{e}	0.07 ±0.04^{a}
**PC**	58.94 ±0.47^{f}	6.59± 0.09^{e}	17.76± 0.19^{f}	18.94 ± 0.2^{f}	4.41 ±0.57^{e}
**BC**	56.64± 0.53^{g}	3.99± 0.06^{f}	15.93 ±0.07^{g}	16.42 ±0.06^{g}	0.8 ± 0.24^{f}
**Bl-C**	42.93 ± 0.2^{h}	6.89 ±0.06^{g}	13.32 ±0.11^{h}	14.99 ±0.13^{h}	0.14 0.02^{a}

WF- white wheat; PF- purple Wheat; BF- blue Wheat, Bl-F- black Wheat, WC- white wheat chapatti; PC- purple Wheat chapatti; BC- blue Wheat chapatti and Bl-C- black Wheat chapatti. All values are expressed as mean ± S.D (n=6). Different letters in the subscripts are significantly different ($p<0.05$).
